# Circulating metabolome in relation to cognitive impairment: a community‐based cohort of older adults

**DOI:** 10.1002/alz.088773

**Published:** 2025-01-09

**Authors:** Yuhui Huang, Xuehui Sun, Qingxia Huang, Qiumin Huang, Xiao Chen, Xiaofeng Zhou, Hui Chen, Jie Shen, Mengyan Gao, Hui Zhang, Huiru Tang, Xiaofeng Wang, Xiaoyan Jiang, Yan Zheng, Changzheng Yuan

**Affiliations:** ^1^ School of Public Health, the Second Affiliated Hospital, Zhejiang University School of Medicine, Hangzhou, Zhejiang China; ^2^ Fudan University, Shanghai, Shanghai China; ^3^ Tongji University, Shanghai, Shanghai China; ^4^ Harvard T.H. Chan School of Public Health, Boston, MA USA

## Abstract

**Background:**

The role of circulating metabolome in cognitive impairment is limited and inconclusive. We aimed to identify plasma metabolites associated with cognitive impairment and evaluate the added predictive capacity of metabolite biomarkers on incident cognitive impairment beyond traditional risk factors.

**Method:**

In the community‐based Rugao Longevity and Ageing Study (RuLAS), plasma metabolome was profiled by nuclear magnetic resonance (NMR) spectroscopy. Participants were classified into the cognitively normal, moderately impaired, and severely impaired group according to their performance in two objective cognitive tests. A two‐step strategy of cross‐sectional discovery followed by prospective validation was applied to identify cognitive impairment‐related metabolites. In the discovery stage, we included 1643 participants (age: 78.9 ± 4.5 years) and conducted multinomial logistic regression. In the validation stage, we matched 68 incident cases of cognitive impairment (moderately‐to‐severely impaired) during the 2‐year follow‐up to 204 cognitively normal controls by age and sex with a 1:3 ratio and conducted conditional logistic regression. Metabolite set enrichment analysis was performed to identify cognitive impairment‐related metabolic pathways. We constructed prediction models for incident cognitive impairment using Lasso regression.

**Result:**

We identified 28 metabolites cross‐sectionally related to severely impaired cognition, among which IDL particle number, ApoB in IDL, leucine, and valine were each prospectively associated with 28%, 28%, 29%, and 33% lower risk of incident cognitive impairment. Based on incident cognitive impairment‐related metabolites, three enriched metabolic pathways (valine, leucine, and isoleucine biosynthesis; valine, leucine, and isoleucine degradation; and aminoacyl‐tRNA biosynthesis) were found. Incorporating 13 metabolite biomarkers selected by Lasso regression into the traditional risk factors‐based prediction model substantially improved the prediction performance on incident cognitive impairment (AUCs: 0.839 vs. 0.703, P <0.001).

**Conclusion:**

The study identified specific plasma metabolites and potential enriched metabolic pathways related to cognitive impairment. Utilizing the selected metabolites substantially improved the prediction performance for cognitive impairment.

